# 2183 Balancing patient-centeredness and patient safety in the hospitals: The case of pain care and patient satisfaction

**DOI:** 10.1017/cts.2018.278

**Published:** 2018-11-21

**Authors:** Olena Mazurenko, Basia Andraka-Christou, Matthew Bair, Areeba Kara, Christopher A. Harle

**Affiliations:** 1 Indiana University School of Medicine; 2 Florida University; 3 Indiana University

## Abstract

OBJECTIVES/SPECIFIC AIMS: This study seeks to understand the relationship between opioid prescribing and patient satisfaction among non-surgical, hospitalized patients. As part of this study, we qualitatively examined challenges in delivering safe and patient-centered care through voices of physicians’, and nurses.’ METHODS/STUDY POPULATION: We collected data through in-person interviews using semi-structured guides tailored to the informant roles. Study participants came from 1 healthcare system located in a mid-Western state. Each interview lasted 30–45 minutes, was audio-recorded with consent, and transcribed for analysis. Two researchers each coded 17 transcripts for discussions around patient-centeredness (including patient satisfaction, patient experiences), and patient safety for hospitalized patients experiencing pain. Analysis followed a general inductive approach, where researchers identified themes related to the research questions using an open coding technique. They discussed and reached consensus on all codes, and extracted several preliminary themes. The analysis was supported by NVivo software. RESULTS/ANTICIPATED RESULTS: The following themes emerged: (1) complex decision-making process to prescribe opioids for hospitalized patients; (2) the role of objective findings in prescribing decisions; (3) bargaining process in prescribing opioids; (4) balancing patient-centeredness and patient safety for selected populations; (5) opioids are the predominant medications for pain care. DISCUSSION/SIGNIFICANCE OF IMPACT: Clinicians’ decision to prescribe opioids for nonsurgical hospitalized patients is based on multiple factors, including patient’s condition, patient’s preference for pain medications, or standard hospital’s pain care regimen. Interventions that improve clinicians’ ability to prescribe opioids may be needed to improve delivery of patient-centered and safe pain care.

